# Surgical Techniques for Lapidus Arthrodesis: Approaches, Indications, and Outcomes

**DOI:** 10.3390/jcm14134591

**Published:** 2025-06-28

**Authors:** Marco Donantoni, Simone Santini, Dario Martinelli, Andrea Marinozzi

**Affiliations:** 1 Department of Orthopaedic and Trauma Surgery, Fondazione Policlinico Campus Bio-Medico, Via Alvaro del Portillo, 200, 00128 Roma, Italya.marinozzi@policlinicocampus.it (A.M.); 2 Research Unit of Orthopaedic and Trauma Surgery, Università Campus Bio-Medico di Roma, Via Alvaro del Portillo, 21, 00128 Rome, Italy

**Keywords:** Lapidus, hallux valgus, I TMTJ instability, first ray arthrodesis

## Abstract

Hallux valgus (HV) is a common forefoot deformity for which numerous surgical techniques have been proposed, with the Lapidus procedure representing a powerful and durable solution, especially in cases of moderate to severe deformities and first ray hypermobility. Initially described in the early 20th century, the Lapidus procedure involves first tarsometatarsal joint (TMTJ) arthrodesis and has undergone multiple modifications over time to reduce complications such as nonunion, malunion, shortening, and recurrence. The technique offers triplanar correction, addressing axial, sagittal, and coronal deformity components. Despite its proven corrective potential, the procedure remains technically demanding, and no universal consensus exists on the ideal fixation method or postoperative protocol. Recent developments in fixation strategies—including crossed screws, locking plates, intramedullary nails, nitinol staples, external fixation, and arthroscopic approaches—have aimed to improve stability, union rates, and the possibility of earlier weight-bearing. This narrative review provides a comprehensive overview of the Lapidus procedure, focusing on surgical indications, technical variants, fixation methods, clinical outcomes, and complications, with the goal of offering practical guidance for optimizing surgical decision-making in various clinical settings.

## 1. Introduction

Hallux valgus (HV) is a deformity of the first ray, characterized by several degrees of severity, for which numerous surgical techniques have been proposed over time. The optimal surgical approach for achieving adequate correction in each specific case remains uncertain [1]. Incorrect selection or execution of the surgical procedure may result in recurrence or complications.

Historically, cases of moderate (hallux valgus angle—HVA < 40° and intermetatarsal angle—IMA > 13°) and severe hallux valgus (HVA > 40° and IMA > 20°) have been associated with poor outcomes, particularly when accompanied by first ray instability and/or pes planovalgus. In such cases, it became evident that a surgical approach capable of both correcting the deformity and stabilizing the first ray was required [2,3].

To this end, arthrodesis of the first tarsometatarsal joint (TMTJ) between the medial cuneiform and the first metatarsal, as well as arthrodesis between the first and second metatarsals, was proposed. This technique later became known as the Lapidus procedure.

The Lapidus procedure was first described by Albrecht [4], Truslow [5], Kleinberg [3], and later popularized by Paul Lapidus [6]. In 1934, Paul W. Lapidus submitted a short report [7] that he would later describe as “personal communication” and “preliminary communication.” It referred to a method of first TMTJ arthrodesis for the treatment of a congenital predisposition toward hallux valgus because of metatarsus primus varus. When Lapidus introduced the procedure, only a single plane of instability, the axial plane, was addressed, with the goal of correcting the increased I–II IMA. The procedure described by Lapidus was introduced for HV correction only in the early 20th century when it was recognized that this condition is a triplanar deformity, leading to technique modifications. Compared with distal metatarsal osteotomies, the Lapidus technique provides greater corrective power because of its more proximal intervention [8,9].

The triplanar deformity involves axial metatarsus varus, sagittal hypermobility, and coronal pronation of the first metatarsal. Increasingly recognized is coronal plane rotation; first metatarsal pronation was found to be present in 87.3% of HV deformities evaluated using weight-bearing computed tomography scans [10].

In addition, first TMTJ hypermobility is now recognized as contributing to both primary and recurrent HV. Unaddressed first metatarsal pronation and hypermobility have been implicated in deformity recurrence and may also negatively affect patient-reported outcome measures (PROMs). The anatomic center of rotation of angulation (CORA) of the HV deformity often lies at the TMTJ. Thus, the triplanar nature of the deformity may be best addressed at the first TMTJ [11].

The alignment of the first metatarsophalangeal joint (MTPJ) is typically maintained by a complex interplay of tendons, ligaments, and bony structures that ensures a proper balance between medial and lateral stabilizing forces. When an imbalance in these forces occurs, the medial structures may become lax while the lateral structures contract, leading to the development of the deformity known as HV. As the deformity progresses, instability of the first ray may develop, driven by these deforming forces on the intrinsic musculature and the loss of the windlass mechanism.

The concept of first ray hypermobility was first introduced by Morton in 1928 [12] and later popularized by Lapidus, who proposed that it contributes to lateral ray metatarsalgia and symptomatic excessive pronation. Instability of the first ray is thought to play a key role in both deformity formation and associated pain. The first ray is critical in weight transfer during the mid-stance and toe-off phases of gait. When unstable, the weight-bearing forces shift laterally to the forefoot, often resulting in callus formation beneath the lesser metatarsal heads, transfer metatarsalgia, hammertoe deformities, or metatarsal stress fractures [13]. The primary site of first ray mobility is the first TMTJ [8]. Normally, the first TMTJ axis is oriented at 45° to the sagittal and frontal planes and parallel to the transverse plane. In HV deformities with first ray pronation, the transverse (mediolateral) plane becomes the dominant axis of instability, followed by the sagittal (dorsoplantar) and frontal (rotational) planes, respectively [2,14].

The traditional Lapidus technique involves both arthrodesis of the first TMTJ and fusion of the bases of the first and second metatarsals [4]. The term “modified Lapidus procedure” was introduced to indicate that arthrodesis was limited to the first TMTJ, without fusion of the first and second metatarsal bases [15]. The introduction of the modified Lapidus procedure was motivated by concerns regarding the excessive rigidity associated with the traditional version. Since its initial description, the technique has been modified by various authors to mitigate potential postoperative complications, particularly the challenge of achieving a stable arthrodesis to reduce the risk of nonunion. Nonunion remains a significant concern, and despite numerous clinical and biomechanical cadaveric studies [1,8,14], the optimal surgical technique and method of fixation for first TMTJ arthrodesis continues to be a topic of debate.

In this article, the authors aim to provide a comprehensive discussion of the Lapidus procedure and its variants, detailing the various techniques and methods of fixation along with their reported outcomes in the literature [16]. This narrative review seeks to expand the existing body of knowledge on the subject and offer guidance in selecting the most appropriate implant for specific clinical scenarios. Given the increasing interest in early weight-bearing protocols and the biomechanical implications of different constructs, a comprehensive overview of the current evidence is needed. This narrative review aims to summarize the key aspects of the Lapidus procedure, including surgical indications, technical variants, fixation methods, clinical outcomes, and complications, in order to provide practical guidance for orthopedic surgeons dealing with complex forefoot deformities.

### Literature Search Strategy

To identify the relevant literature, we conducted a nonsystematic search using PubMed and Scopus. The search included the following terms and Boolean operators: (“Lapidus” OR “first tarsometatarsal arthrodesis”) AND (“hallux valgus” OR “first ray instability”) AND (“fixation” OR “screws” OR “plates” OR “outcomes”). We considered peer-reviewed studies published in English primarily between 2000 and 2023, although earlier landmark publications were also included for historical context. Inclusion criteria were clinical studies, biomechanical analyses, reviews, and relevant technical notes. Editorials, letters, and case reports were excluded. Additional sources were identified by manually screening the references of selected articles.

## 2. Indications and Contraindications

The main indications and contraindications for the Lapidus procedure highlighted in the literature are summarized in Table 1 and remain a topic of ongoing debate [3].

Over the years, it has become evident that distal metatarsal osteotomies are insufficient for correcting severe hallux valgus, particularly in cases where the IMA exceeds 15°. For such patients, proximal first metatarsal osteotomies or first TMTJ arthrodesis are preferred. Another key indication for this surgical option is TMTJ instability [17]. While alterations in the IMA are easily identified clinically and measurable through simple weight-bearing radiographs, accurately assessing TMTJ instability is more challenging. Plantar gapping of the first TMTJ on a lateral weight-bearing radiograph is considered a reliable sign; however, clinical evaluation is highly subjective and difficult to quantify objectively [18,19]. Another complex situation where this procedure has demonstrated effective corrective capabilities and high satisfaction rates among both surgeons and patients is in cases of HV recurrence [20,21]. Additionally, when first TMTJ degenerative or posttraumatic osteoarthritis is present alongside HV, the Lapidus arthrodesis addresses both issues simultaneously. In cases of juvenile HV, the growth status of the epiphyses must be considered, particularly as the epiphysis at the base of the first metatarsal contributes approximately 50% to the overall length of the first ray. For this reason, the Lapidus procedure can only be performed when the growth plates are closed, as open growth plates are considered a contraindication for this surgery. Other absolute contraindications include acute or chronic infections and Charcot arthropathy with or without uncontrolled diabetes. Smoking is regarded as a relative contraindication, as it appears to negatively affect the fusion rate. Additionally, even with meticulous preparation of the joint surfaces, the procedure inevitably results in a shortening of the first ray [22,23]. Therefore, in cases of a preexisting short first metatarsal, the need for interpositional bone block should be anticipated. Another critical factor to evaluate is the patient’s level of athletic activity. Although the available literature on this subject is limited and inconclusive, existing studies highlight a higher risk among athletes (30%) compared with sedentary patients (25%) of not returning to preoperative activity levels [24].

## 3. Preoperative Preparation

Before performing the Lapidus procedure, it is essential to collect a detailed medical history, perform a thorough clinical examination, and conduct a comprehensive radiographic diagnostic work-up.

### 3.1. Clinical Evaluation

Clinical evaluation of the foot consists of a detailed medical history and clinical examination. This includes the patient’s general health, previous related surgeries, occupation, activities of daily living, level of sporting activity, and especially a detailed description of the patient’s pain.

The clinical examination must include a comprehensive assessment of the hindfoot, midfoot, and forefoot, beginning with a gait analysis. The patient is examined both standing and supine to identify any structural abnormalities (such as posterior tibial tendon dysfunction, ankle instability, and gastrocnemius contracture or Achilles tendon alterations) and deformities (including foot deformities such as flatfoot or first ray deformities such as hammer or claw toe) or the presence of skin alterations (e.g., plantar keratoses, scars, or ulcers). The first ray, in addition to being evaluated for the deviation associated with HV, must be assessed for both pain and mobility at the MTPJ and at the first TMTJ. Shibuya et al. [25] reported significantly increased first ray motion in patients with HV deformity compared with those without it, with a mean difference of 3.62 mm in the sagittal plane. The first to describe a method for evaluating the instability of the first TMT joint was Morton [26]. According to his approach, with the patient seated, the knee flexed, and the ankle in a neutral position, one hand stabilizes the lateral four metatarsals while the other hand moves the first ray, both dorsoplantar and mediolateral. Mobility is then compared with the contralateral side [27]. As previously mentioned, obtaining an objective evaluation of this characteristic is challenging and largely depends on the surgeon’s experience. However, this method is subjective and does not allow for an objective quantification of instability [28]. Another option involves the use of external calipers, such as the Klaue device [29], which has demonstrated that the first metatarsal head is elevated by an average of 9.3 mm (±1.9 mm) in patients with hallux valgus, compared with 5.4 mm (±1.4 mm) in patients without the deformity. A more recent and promising approach involves 3D reconstruction based on CT imaging [30], which has the potential to objectively quantify hypermobility of the first TMTJ. As suggested by Schmid et al. [17] “pain at the medial Lisfranc joint, indicating Lisfranc arthritis, general hyperlaxity, an older sedentary patient, and recurrence after previous hallux valgus correction support a Lapidus procedure. Pain in the first MTPJ indicating first MTPJ arthritis, an athletic activity, and nicotine abuse should lead to consideration of other options”.

### 3.2. Imaging Work-Up and Surgical Planning

Imaging evaluation initially relies on weight-bearing radiographs of the foot in the dorsoplantar and lateral views, anteroposterior and lateral radiographs of the ankle, as well as mortise views. Additionally, the Saltzman projection is used to assess hindfoot alignment [31].

The evaluations and parameters measured from these images are crucial for diagnosis and highly valuable for surgical planning. The initial assessments focus on radiographic parameters used to quantify the severity of HV: hallux valgus angle (HVA), intermetatarsal angle (IMA), distal metatarsal articular angle (DMAA), and interphalangeal angle (IPA) [32,33,34,35,36]. Normally, the first TMTJ is slightly medially oriented, but in some cases, it may exhibit excessive medial inclination, leading to a metatarsus primus varus deformity and increasing the likelihood of HV [5]. Additional important considerations include the position of the sesamoids, the congruence of the first MTPJ, and the presence of arthritis or arthrosis in the first TMTJ, first MTPJ, or Lisfranc joint. As previously mentioned, first ray hypermobility must also be assessed. In this context, bony hypertrophy of the second metatarsal head or cortical thickening of the first and second metatarsals may indicate potential first TMT joint instability, as suggested by Morton [9], although this was not confirmed by Grebing and Coughlin [37]. A concept introduced earlier regarding TMTJ instability involves the presence of plantar gapping on lateral radiographs. This is observed in approximately 20% of moderate to severe HV cases or in instances of first TMTJ instability [18,19,38]. According to Schmid et al. [17], “Severe HV with an IMA greater than 15° and arthritis of the first TMTJ on dorsoplantar radiographs supports a Lapidus procedure. In contrast, a short first metatarsal, an open growth plate, and arthritis of the first MTPJ are contraindications.” Additionally, in approximately 8% of patients, the presence of an os intermetatarseum may be observed [18]. In most cases, this is associated with increased rigidity of the first TMTJ and can hinder proper soft tissue healing and restoration of the correct IMA when a distal metatarsal osteotomy is performed. In such cases, it may indicate the need for a Lapidus procedure. In some cases, it may be necessary to use CT scans to better evaluate the architecture of the bony components. A single photon emission CT (SPECT-CT) scan is particularly useful for defining the extent of activity of degenerative processes in the adjacent joints and, if necessary, to plan surgical intervention for these as well [39]. Magnetic resonance imaging (MRI) is helpful in identifying soft tissue abnormalities. In cases of HV associated with flatfoot, posterior tibial tendon dysfunction is often observed.

### 3.3. Variants of the Technique

As previously mentioned, the original Lapidus technique involves not only arthrodesis of the first TMTJ but also fusion between the bases of the first and second metatarsals, and it is typically performed alongside a distal soft tissue procedure [4]. The key distinction between the original Lapidus and modified Lapidus procedures lies in the first-to-second ray arthrodesis, which is included in the original technique but omitted in the modified version [15]. The modified Lapidus was initially developed to address concerns related to excessive stiffness and overload of the first ray—particularly at the level of the sesamoids—that were observed with the conventional approach. Over time, however, these two procedures have evolved into clearly distinct techniques, each with its own specific indications and clinical outcomes. In a cadaveric study [40], the sagittal plane motion of the first ray was measured at 7.45 ± 1.82 mm prior to fusion of the first TMTJ. Isolated first TMT joint arthrodesis using a crossed screw technique reduced this sagittal motion to 4.41 ± 1.51 mm, and the addition of middle cuneiform fixation further decreased it significantly to 3.12 ± 1.06 mm. These findings suggest that fixation between the first and second rays can be advantageous, and potentially necessary, in cases where excessive sagittal motion persists after isolated first TMT joint fusion. In recent years, the modified Lapidus procedure is usually the first-line approach, as it allows for a small degree of sagittal motion within the medial column, which can help unload the sesamoids in the setting of a rigid metatarsal. However, surgeons remain mindful of avoiding excessive mobility between the first and second metatarsals or between the medial and middle cuneiforms, as such instability may contribute to the recurrence of deformity, particularly through increasing varus deviation in the first metatarsal as it drifts medially away from the second metatarsal. After completing the modified Lapidus procedure, a stability assessment as described by Valderrabano et al. [3] can be performed to detect residual instability. Under fluoroscopy, the surgeon dynamically compresses the first web space using the thumb and index fingers. If the IMA increases, this indicates first-to-second ray instability. In such cases, the “original” Lapidus procedure is indicated, and the stability of the first ray is then re-evaluated using the same test after internal fixation has been completed.

## 4. Surgical Techniques

### 4.1. Patient Set-Up

The patient is placed in a supine position on the operating table, with the foot positioned at the edge of the bed. If needed, a bolster can be placed under the ipsilateral buttock to achieve optimal foot rotation. A well-cushioned pneumatic thigh tourniquet is secured around the designated thigh. The procedure is commonly carried out under spinal anesthesia, though regional techniques, such as a popliteal or ankle block, may also be utilized. The surgical site is prepared and sterilized up to the knee, following standard aseptic protocols. After exsanguination of the limb, the tourniquet is inflated to a pressure of 100 to 150 mm Hg above the patient’s systolic blood pressure.

### 4.2. Classic Approach: Incision, Preparation of Joint Surfaces [3,17,41]

The skin incision is located at the dorsomedial level, starting approximately 1 cm proximal to the first TMT joint and directed distally toward the first MTP joint, extending to reach the first interphalangeal joint.

The interval is between the tibialis anterior (TA) tendon and the extensor hallucis longus (EHL) tendon. Depending on the author’s preference, either the proximal or distal portion is addressed first. If attention is directed first to the metatarsal head [42], the deep fascia is incised in line with the incision, raising the dorsal and plantar flaps. The EHL is identified and retracted laterally. The dorsomedial digital branch of the medial cutaneous nerve and the medial branch of the common digital artery, which may be at risk, are identified and protected.

A Y-shaped or longitudinal capsulotomy of the first MTPJ is performed. Using sharp dissection, the capsule is freed dorsally, laterally, and plantarly to access the lateral MTP structures and sesamoids. The medial eminence is resected with a standard cut of 2 to 4 mm in width, superior more than inferior, and without violating the medial sesamoid groove. Lateral release is performed if indicated. Distal soft tissue release is a crucial step to allow correction of the IMA. This includes releasing the capsule, adductor tendon, lateral collateral ligament, and transverse metatarsal ligament lateral to the first metatarsal head.

Proximally, the joint capsule of the first TMTJ is approached by retracting the EHL tendon laterally. Care must be taken to protect the dorsal cutaneous nerves. The incision is deepened in the same plane straight down to the periosteum at the base of the first metatarsal, and the joint is opened dorsally and medially.

The dorsal tarsometatarsal ligaments at the TMTJ are resected, and periosteal flaps are raised circumferentially around the first TMTJ. At this point, the first TMTJ is exposed using two Hohmann retractors to protect the dorsal and plantar soft tissues during the preparation of the joint surfaces. The cut on the first metatarsal articular surface is made along its border, parallel to the base of the first metatarsal and perpendicular to its long axis. The medial cuneiform articular resection is performed perpendicular to the axis of the second metatarsal bone in the coronal plane, thereby removing a lateral wedge of the medial cuneiform. To prevent shortening of the first ray, it is crucial to start the cuneiform cut within the cuneiform cartilage area. The wedge resection is performed, directing the saw towards the II metatarsal. If plantarflection of the first ray is needed, the cut can be executed biplanarly by removing more bone laterally than medially and more on the plantar side than dorsally. At this point, on the first TMTJ, the subchondral bone of both exposed articular surfaces is treated with an osteotome, and multiple 1.5 mm drill holes are created to promote arthrodesis. The correction of the deformity and preservation of the first ray biomechanics are achieved by shifting the joint from a dorsomedial to a plantar-lateral position. This is accomplished through a maneuver that includes first metatarsal adduction, supination, and simultaneous maintenance of neutral plantarflexion, combined with axial compression of the MTPJ. Only in this way can pronation be effectively corrected. It is important to note that there is often a tendency to undercorrect metatarsal plantarflexion, which can lead to transfer metatarsalgia of the second ray. Once the correct alignment and rotation are achieved, the desired position is temporarily fixed with Kirschner wires. A clinical and fluoroscopic evaluation of the result is then performed. If satisfactory, definitive fixation is carried out, which can be achieved using a variety of fixation devices.

### 4.3. Methods of Fixation

Over the years, various types of fixation devices have been proposed, without a definitive consensus on which is superior. The choice of the ideal fixation method begins with the surgeon’s awareness that the skin, particularly on the dorsal side, is especially vulnerable, making implant prominence and subsequent patient discomfort a potential concern. When focusing specifically on the type of fixation, it is also essential to consider whether a traditional Lapidus procedure is required (in cases of instability between the first and second ray) and to select the most appropriate fixation device to ensure a higher union rate and enable early mobilization.

#### 4.3.1. Fixation with Crossed Screws

The use of crossed screws across the joint has traditionally been the most common and cost-effective method of fixation. However, some authors have raised concerns that the compressive forces generated by this technique may be insufficient, as optimal compression at the first TMT joint is estimated to require between 80 and 100 N [41]. This limitation implies that crossed screw constructs cannot be employed indiscriminately. According to other authors, fixation with two crossed 3.5 mm lag screws generally provides adequate stability [17]. Typically, the first screw is placed from distal to proximal. It is broadly agreed in the literature that a notch should be created at the entry point of the distal screw to prevent dorsal cortical fractures of the proximal first metatarsal. Without this notch, the screw head may act as a lever, elevating the dorsal cortex when it engages the bone. To create the notch, a burr or a triangular configuration of six 2.5 mm drill holes has been proposed, and the bone is carefully removed. Given that the medial cuneiform measures approximately 1.5 cm in width, precise drill alignment is essential to ensure correct screw placement and to avoid breaching the intercuneiform joint. Drilling parallel to the medial border of the foot assists in maintaining alignment along the axis of the cuneiform. A 3.5 mm gliding hole is typically prepared first. If more lateral correction of the first metatarsal is desired, the 2.5 mm drill can then be angled slightly more medially within the cuneiform compared with the gliding hole. The second screw is introduced from proximal to distal, targeting the lateral plantar cortex of the first metatarsal. It is important to consider that the screws should be positioned so they cross distal to the fusion site rather than within it, thereby providing added rotational stability. Furthermore, dorsiflexion of the first metatarsophalangeal joint during screw insertion enhances compression at the arthrodesis site by placing tension on the plantar fascia.

#### 4.3.2. Plate Fixation

An alternative method of fixation involves the use of a plate, which can be positioned dorsally, dorsomedially, medially, or along the plantar aspect of the joint. In recent years, it has become increasingly clear that the combination of a single dorsal compression screw with a medial plate offering additional compressive capacity enhances the biomechanical properties of the construct. This configuration improves overall stability and provides superior resistance to loading forces in both the sagittal and transverse planes. However, a cadaveric study comparing dorsomedial and plantar plating techniques reported some advantages of the plantar approach. Specifically, it was associated with fewer injuries to high-caliber subcutaneous veins and a lower risk of damaging superficial nerves, such as the saphenous and superficial fibular nerves. Neither technique affected the insertion of the tibialis anterior tendon. Both are considered safe and established procedures, but plantar plating may offer additional benefits and represents a reliable option in the surgeon’s armamentarium [43]. In addition, another anatomical study identified a “safe zone” for plantar plate placement, minimizing the risk of injury to surrounding structures, such as the tibialis anterior and peroneus longus tendons. This supports the feasibility of plantar plate application without the need for significant contouring and with a low risk of soft tissue irritation [44].

#### 4.3.3. Plating Plus a Lag Screw

In cadavers, data demonstrated that the time required to reach 50% of peak compression in a “plate-with-lag-screw” configuration was approximately threefold that of a construct using a plate alone [45]. A clinical cohort study involving 59 feet from 58 patients reported that this fixation approach allowed for protected full weight-bearing at an average of seven weeks postoperatively, achieved a bony union rate of 98.31%, and resulted in a patient satisfaction rate of 94.12% [46]. In addition to plate placement, incorporating a compression screw across the first TMT joint may enhance construct stability, promote a high union rate, and facilitate early postoperative mobilization. Comparable biomechanical findings have been observed when varying the orientation of the compression screw.

Among the various plate designs available, although plantar plating shows several advantages, the dorsomedial low-profile locked plate is the most commonly used. It is anatomically contoured to extend from the dorsal aspect of the medial cuneiform to the medial base of the first metatarsal. Many systems are designed to accommodate olive-tipped K-wires as an adjunct to enhance compression across the arthrodesis site. The surgical sequence typically begins with screw placement through the proximal cuneiform plate holes [16]. A compression screw is then inserted distally into the metatarsal metaphysis, positioned within the dynamic slot to further augment compression at the fusion site. The remaining plate holes are subsequently filled with locking or nonlocking screws, with the choice tailored to the surgeon’s preference and the patient’s bone quality. Additional advantageous features available in some systems include a plantar locking arm, which provides improved resistance to tensile forces at the joint, and a precision guide that facilitates the insertion of a dorsal compression screw while minimizing the risk of interference with the locking plate screws [41].

#### 4.3.4. Adjunct Procedures

Additional procedures may be required. If the first MTPJ remains incongruent following Lapidus arthrodesis, a Chevron osteotomy can be performed. The Akin osteotomy is the most commonly performed procedure for correcting an abnormal IPA and proximal phalangeal pronation. As previously mentioned, the Akin osteotomy can be carried out either by extending the surgical approach distally or through a separate incision. Typically, a closing wedge osteotomy is performed at the base of the proximal phalanx and stabilized using a 2.5 to 3.0 mm cannulated compression screw. In patients with symptomatic metatarsalgia or an abnormal metatarsal parabola, Weil osteotomies may be indicated. A Weil osteotomy of the lesser metatarsals can also help restore length to a shortened first ray. When necessary, additional deformities, such as pes planovalgus, posterior tibial tendon dysfunction, or gastrocnemius contracture, should be concurrently addressed as part of a comprehensive HV correction strategy.

## 5. Alternative Techniques for Lapidus Procedures

### 5.1. Staple Fixation

Nitinol compression staples represent an additional fixation option available to surgeons. Their main theoretical advantage lies in the ability to maintain continuous compressive forces after implantation, in contrast to screw fixation, where some loss of compression typically occurs immediately after insertion [47]. Although there is limited evidence in the literature supporting the efficacy of nitinol staples compared with other fixation methods, they are predominantly employed as adjunctive tools, particularly to address or prevent plantar gapping at the arthrodesis site.

### 5.2. Intramedullary Fixation

The discomfort experienced by patients at the level of the dorsal skin due to prominent hardware has led to the development of intramedullary nailing systems. From a biomechanical standpoint, the intramedullary nail is capable of withstanding higher loads across the fusion site, while also limiting implant migration during the healing process and providing uniform compression across the first TMT joint. Ultimately, this configuration supports the possibility of earlier weight-bearing. Compared with other fixation methods, the intramedullary nail can counteract forces across three planes, including rotational, dorsiflexion, and mediolateral bending stresses. A notable feature is the inclusion of a calibrated compression device, which allows the surgeon to accurately assess when optimal compression has been achieved. The nail is available in lengths ranging from 38 to 60 mm and is equipped with threaded pegs for bicortical fixation, with the threads engaging the nail proximally and then distally following compression.

### 5.3. External Fixation

The use of external fixation in the Lapidus procedure represents another valuable option, particularly appealing because it allows for immediate postoperative weight-bearing, leaves no internal hardware once the device is removed, and, when properly executed, is associated with high fusion rates. Currently, this technique is employed primarily in revision cases, particularly when previous procedures have been complicated by infection or osteomyelitis, given that available data in the literature remain limited and somewhat conflicting [48].

In fact, a study with over 100 patients reported a mean time to unassisted weight-bearing of 13.1 days, an average fusion time of 5.3 weeks, and fixator removal at approximately 5.5 weeks postoperatively [49]. Notably, these reports documented no cases of nonunion or delayed union, with only a single case of pin tract infection successfully managed with oral antibiotics and local wound care. Conversely, another study with a considerably smaller sample size [50], including 11 Lapidus procedures performed with a rail external fixator, reported a high incidence of complications. These included premature frame removal, the need for intravenous antibiotic therapy, and two cases of nonunion, underscoring the variability in outcomes reported in the current literature.

### 5.4. Arthroscopic Lapidus Arthrodesis

Another option for performing the Lapidus procedure is the arthroscopic technique. First described by Lui and colleagues in 2005 [51], it was recommended that this approach be reserved for surgeons experienced in small joint arthroscopy because of its considerable technical demands. From the outset, the technique has generated interest, particularly because it allows improved visualization of the plantar and lateral aspects of the joint. Additionally, when performed by skilled hands, arthroscopy enables more precise removal of the articular cartilage, while preserving the subchondral bone and reducing the risk of excessive shortening of the first ray. Ultimately, it shares the benefits common to minimally invasive procedures, including smaller and more cosmetically favorable scars, reduced postoperative swelling and pain, and a lower risk of infection [52,53].

The technical description of the procedure [51] includes the use of a 2.7 mm, 30° arthroscope, a small periosteal elevator, an arthroscopic osteotome, and an arthroscopic awl. A 21-gauge needle is first inserted into the joint, which is then distended with 2–3 mL of sterile normal saline. Two arthroscopic portals are created, one plantar–medial and one dorsomedial to the joint. As previously noted, after visualizing the joint with the arthroscope, the articular cartilage is debrided using the osteotome and periosteal elevator, leaving the subchondral bone intact. Microfracture of the subchondral surface is then performed using arthroscopic awls. Once the intermetatarsal angle is reduced and mild plantarflexion achieved, a 4.0 mm cannulated screw is inserted from the proximal dorsal aspect to the distal plantar aspect to compress the joint. If a traditional Lapidus procedure is required, a second 4.0 mm positioning screw is placed from the base of the first metatarsal to the base of the second metatarsal and is typically removed after approximately 12 weeks. Patients are kept nonweight-bearing for an extended period, up to 12 weeks, following this approach. Arthroscopy of the first TMTJ offers a promising minimally invasive alternative for the Lapidus procedure. Lui and colleagues [51,52] have reported single case studies demonstrating the use of arthroscopy in both primary and revision arthrodesis. Michels and colleagues [54] performed arthroscopic Lapidus fusions on five patients, reporting improvements of 25.6° in the hallux valgus angle, 10.6° in the intermetatarsal angle, and minimal shortening of the first ray (limited to 2.7 mm). All five patients achieved successful fusion.

## 6. Postoperative Care

Physical therapy, lymphatic drainage, cryotherapy, and leg elevation are recommended as part of the postoperative management following surgery. An area of variability among surgeons is the need for postoperative immobilization using boots or casts, as well as the appropriate duration for their use. While casting appears to offer greater reassurance in maintaining bone alignment and optimizing compression across the fixation construct, it may hinder the initiation of early physical therapy targeting the first MTPJ and hindfoot, which is considered beneficial in promoting functional recovery. There is currently no consensus in the literature regarding the optimal postoperative protocol following the Lapidus procedure. Even among authors utilizing the same fixation methods, notable differences in postoperative management are reported. These variations largely reflect both the wide range of fixation devices available and the well-recognized risk of complications, particularly related to difficulties in achieving successful joint fusion. In this context, several studies have explored whether allowing immediate or very early weight-bearing was described to have no adverse effects on alignment or the rate of osseous union relative to more traditional approaches [55,56,57]. However, given the limited data available and the lack of clear evidence supporting the superiority of early weight-bearing protocols, most surgeons today still tend to favor more conservative strategies with cautious and gradual progression to full weight-bearing [23,58]. Most investigators recommend nonweight-bearing in the first 6 to 8 weeks after the procedure. Some surgeons keep patients nonweight-bearing for 4 weeks and a 10 to 20 kg partial weight-bearing for the next 4 weeks [59]. Other protocols allow for early weight-bearing using a stabilizing walker, typically with 15 kg of partial weight-bearing, during the first 6 weeks. After this period, patients are generally advised to transition to full weight-bearing while using a more flexible stabilizing shoe for an additional 6 weeks [16]. In most cases, gradual progression to full weight-bearing is initiated after 8 weeks, typically following dorsoplantar and lateral radiographic evaluation. This aspect of postoperative care also shows variability among surgeons; when partial weight-bearing is permitted at 4 or 6 weeks, radiographs are generally obtained at these time points and repeated prior to advancing to full weight-bearing. These locking plates with compression screws have become increasingly favored in recent practice, and biomechanical studies did not show different rigidity as compared to a screw construct with the numbers available [60]. In this context, biomechanical investigations also demonstrate that plantar plates provide comparable or superior construct stability to dorsomedial plates or screw-only constructs. A study evaluating medio–plantar versus purely plantar plates reported equivalent bending stiffness and load-to-failure performance [61]. This aligns with a recent systematic review suggesting that plantar locking plates combined with compression screws offer enhanced mechanical stability and potentially earlier weight-bearing capabilities [62].

## 7. Complications

Potential complications following the Lapidus procedure include nonunion or malunion, delayed union, prominent hardware, excessive shortening of the first ray, undercorrection or recurrence of the deformity, overcorrection with negative intermetatarsal angle or hallux varus, and nerve injury [16,63].

### 7.1. Nonunion

Historically, the Lapidus procedure was associated with a high nonunion rate, reported between 6% and 12% [22,64]; however, in recent years, several studies have reported nonunion rates approaching zero [65,66]. In simultaneous bilateral Lapidus, the rate even rises up to 33% [23].

Three main factors are consistently identified as concerns in achieving successful outcomes: the technique of joint preparation, the type of fixation construct employed, and the allowance of early weight-bearing. These are considered surgeon-related factors. In addition, patient-related factors must be considered, including overall health status, such as the presence of diabetes, smoking habits [21], and vitamin D and calcium levels. Patient compliance also remains a constant and critical consideration. Joint preparation involves planal resection of the joint surfaces or curettage. Planal resection provides an excellent bleeding bone surface that supports healing, but it can be challenging to achieve optimal alignment and carries the risk of excessive first ray shortening. Curettage was introduced as an alternative to address some of these limitations. However, concerns with curettage include the potential for increased nonunion rates, as it aims to preserve the subchondral plate. Prissel and colleagues reported a superior union rate with planal resection (97.8%) compared with curettage (91.5%) [66]. Despite this, curettage has still been associated with relatively low nonunion rates [59]. Patel and colleagues reported a 5.3% nonunion rate in a series of 227 patients treated with curettage, and a larger study involving 599 patients showed a comparable nonunion rate of 5%.

The method of fixation used for Lapidus arthrodesis has continued to evolve to minimize the risk of nonunion. Donnenwerth and colleagues conducted a systematic review on the use of curettage combined with crossed screw fixation and reported a nonunion rate of 5% [67]. More recently, there has been a trend toward more stable fixation constructs to facilitate earlier weight-bearing. Studies have shown that interfragmentary screws combined with locking plates provide superior stability compared with crossed screws or interfragmentary screws with nonlocking plates [66]. In a systematic review by Crowell and colleagues [68], which analyzed 443 arthrodesis cases, early weight-bearing was defined as commencing within two weeks postoperatively; the reported overall nonunion rate was 3.61%. In a multicenter study by Ray and colleagues, most patients were permitted full weight-bearing as tolerated in a walking boot on the day of surgery, with a reported symptomatic nonunion rate of just 1.6% [69]. Another multicenter study involving 80 patients reported a 100% union rate, with a mean time to weight-bearing of 14.8 days [65].

Initially, it was believed that radiographically detected nonunion would also carry significant clinical consequences; however, it now appears that probably only 25% to 50% of patients with radiographic nonunion present with associated clinical symptoms [38]. Revision surgery should be considered only if the patient is symptomatic. The largest published case series on this topic, by Hamilton and colleagues, evaluated union rates following revision Lapidus arthrodesis [70]. Seventeen cases of revision bone block TMT arthrodesis were analyzed. Bone stimulators were applied immediately postoperatively in 13 patients. The postoperative protocol included nonweight-bearing for six weeks in a short leg cast, followed by progression to a walking boot for four weeks if radiographic signs of consolidation were observed. Patients were typically transitioned to regular footwear for an average of 10 weeks. Of the total cohort, fourteen patients (82%) achieved union, while three patients (18%) experienced recurrent nonunion. Among these, two patients remained symptomatic at the time of study completion, while one underwent further revision surgery, ultimately achieving union. Notably, this study identified smoking as a statistically significant predictor of nonunion. In another study assessing nonunion rates after modified Lapidus arthrodesis, Patel and colleagues [59] briefly described revision procedures performed in seven cases of nonunion following Lapidus bunionectomy. All revision cases utilized autogenous bone grafts harvested from the iliac crest, distal tibia, or calcaneus. Of these, six patients achieved successful union, while the remaining patient was asymptomatic and did not require further surgical intervention.

### 7.2. Malunion and Recurrence

Malunion following a Lapidus procedure can manifest in various forms. In some cases, malunion results from suboptimal positioning of the fusion, reflecting the technical challenges inherent in this procedure. According to some authors [23], the relatively high incidence of failure can be attributed both to the procedure’s technical complexity and to the associated surgical learning curve. Additionally, delayed weight-bearing may limit the surgeon’s ability to detect intraoperative errors in positioning. Many of these patients have developed a preexisting triplanar deformity even before undergoing surgery, making it not uncommon for certain aspects of the deformity to recur postoperatively. The primary goal of the procedure is to correct the congenital and acquired deformities and to provide a stable, long-lasting construct. Malunion can present clinically as recurrence or undercorrection of the original deformity, elevation or excessive shortening of the first metatarsal, overcorrection, excessive plantarflexion of the first metatarsal, or failure to achieve adequate correction in the frontal plane. The most frequent form of malunion following a Lapidus procedure is, in fact, the recurrence of the original deformity. Reported recurrence rates vary depending on the surgical technique used and range from 3.2% to as high as 60% [69,71]. This complication can arise from multiple contributing factors. However, failure to achieve complete correction and solid fusion at the first TMTJ—particularly when accompanied by a persistently widened IMA—may constitute a true malunion. Recurrence can occur particularly in cases where fixation is inadequate or when multiple components of plantar hypermobility are insufficiently addressed, situations where a true Lapidus procedure would have been more appropriate than a modified Lapidus [72]. As previously mentioned, one of the most common yet often underrecognized causes of recurrence is inadequate derotation (supination) of the first metatarsal.

### 7.3. Shortening

Shortening of the first metatarsal typically results from flat saw cuts or closing wedge resections, where excessive bone has been removed. The literature reports an average shortening of approximately 5 mm following first TMTJ fusion [22,73]. Generally, shortening of less than 5 mm, when combined with correct positioning, does not lead to clinical problems. For shortening between 5 and 10 mm, slight plantarflexion of the first metatarsal is usually sufficient to prevent complications. In cases of shortening greater than 10 mm, shortening metatarsal osteotomies (such as Weil osteotomies) of the second and third metatarsals may be required to prevent transfer metatarsalgia. When shortening exceeds 20 mm, reconstruction of the first ray with a tricortical bone graft is typically necessary to adequately restore length [20]. First metatarsal shortening is associated with an overload of the lesser metatarsals and can lead to metatarsalgia [72], occurring in up to 10% of cases [64]. To avoid this complication, it is critical to perform precise joint preparation and avoid excessive removal of bone wedges. The interposition of the resected medial eminence can help prevent significant shortening, especially in cases where the metatarsal is already relatively short or when too much bone has been resected [73]. To further minimize the risk of shortening, intentional plantarflexion of the first metatarsal or the use of a bone graft at the arthrodesis site has been suggested. However, surgeons must be aware of the limitations of plantarflexion, as excessive plantarflexion can lead to overload and pain at the sesamoids. Moreover, increased plantarflexion may result in hallux dysfunction because of compensatory extension at the metatarsophalangeal joint and extensor hallucis longus contracture, which can further exacerbate plantarflexion of the metatarsal. Bone grafting at the fusion site can reduce the original shortening by approximately 50% [22,73]. Therefore, the most effective strategy to prevent shortening is to perform a rotational first TMTJ fusion, removing only the joint surface while preserving the natural curved shape of the joint.

## 8. Results and Outcomes

### 8.1. Clinical Outcomes

The wide variety of available fixation devices has led to numerous comparative studies. Most recent publications focusing on the Lapidus procedure have centered on locking plate technology.

In a series of 128 patients (144 feet), Wilde and colleagues [74] evaluated the Lapidus procedure using polyaxial locking plates and reported a union rate of 98.6%.

Sorensen et al. [75] achieved a 100% union rate using an interfragmentary screw combined with dorsomedial locking plate fixation and demonstrated that full weight-bearing ambulation was possible as early as two weeks postoperatively.

DeVries and colleagues [76] performed Lapidus procedures on 143 patients, comparing crossed screws to dorsomedial locking plates without interfragmentary compression. They observed a union rate of 89.4% in the crossed screw group and 98.5% in the locking plate group. Additionally, the locking plate group achieved full weight-bearing at an average of 7.8 weeks, compared with 8.8 weeks in the crossed screw group.

Saxena et al. [77] evaluated 40 patients who underwent the Lapidus procedure using either crossing lag screws or a plantar lag screw with locking plate fixation. While they found no significant difference in nonunion rates between the two groups, the locking plate group was able to initiate weight-bearing at four weeks, compared with six weeks in the crossing screw group.

In a recent retrospective multicenter study [10], a biplanar locking plate (BLP) was compared with crossed screw fixation in 130 patients, with 65 patients in each group. This study observed that the BLP system was associated with greater improvements in radiographic hallux valgus parameters compared with the crossed screw fixation technique. However, the clinical significance of these findings remains unclear, as complication and reoperation rates were similar between the two groups.

In another retrospective study [78], the aim was to compare complication rates, as well as the degree and maintenance of angular correction, between a dorsomedial locking plate combined with an intercuneiform compression screw construct and the traditional crossed solid screw fixation construct. A total of 64 feet in 56 patients were included in the study, with 32 cases in each group. Overall, the dorsomedial locking plate plus intercuneiform compression screw construct demonstrated superior maintenance of IMA correction at midterm follow-up compared with the traditional crossed screw construct. Both groups, however, showed similar fusion rates at 10 weeks, as well as comparable rates of complications.

### 8.2. Lapidus and Return to Sport

Performing a Lapidus procedure in the athletic population, particularly elite athletes, remains a subject of debate [79]. The Lapidus procedure is widely regarded as technically demanding, with a relatively high rate of complications, including nonunion, malunion, shortening, elevation, progression of midfoot arthritis, transfer metatarsalgia, neurovascular compromise, hematoma, and hardware-related issues [80,81]. The main concerns are the risk of failure and, even in cases of successful fusion, the residual stiffness resulting from arthrodesis of the first TMT joint. Unfortunately, very few studies provide objective data to help guide surgeons in selecting between a proximal osteotomy and first TMT arthrodesis in this population.

In 2001, McInnes and Bouche [24] published a retrospective study on the outcomes of modified Lapidus arthrodesis, reporting both subjective and objective findings. The study included 32 feet in 25 patients, with an average follow-up of 39 months. The procedure was performed to treat moderate to severe hallux valgus associated with first ray hypermobility. Subjectively, 78% of patients rated the surgery as “completely” or “very” effective; however, the return-to-activity rate among athletes was poor (30%). Objective findings revealed an average shortening of the first metatarsal by 3.4 mm, with mean postoperative first MTP joint dorsiflexion measuring 62.6°. Complications included five nonunions and two delayed unions. No significant differences in outcomes were observed between athletes, active patients, and sedentary patients.

More recently, MacMahon et al. [81] reported more promising results, suggesting that the modified Lapidus procedure is a viable option for athletes. Their retrospective study included a cohort of 48 athletes with an average follow-up of 2.8 years. The procedure was performed for hallux valgus deformity with associated first ray hypermobility. This study reported only subjective outcomes: 81% of patients were satisfied with their return to activities, and 80% were able to resume their previous sports. These data were based on two self-administered postoperative questionnaires. Complications were reported in 15% of patients, ranging from hematoma requiring surgical evacuation to transfer metatarsalgia. Notably, no objective outcome measures were evaluated or reported. The two studies show a substantial difference in return-to-sport rates, which may be related both to the method used to collect these data and to the fact that the second, more recent study likely reflects improvements in surgical technique and fixation methods.

## 9. Conclusions

The Lapidus procedure is a powerful and durable option for correcting moderate to severe hallux valgus and/or first ray hypermobility. Unfortunately, it is a technically demanding procedure that carries a risk of complications, and no universally effective strategies have yet been identified for every clinical scenario. While some authors advocate for early weight-bearing when using crossed lag screws and locking plates, the consensus remains that larger prospective and randomized clinical trials are necessary to more precisely determine the appropriate duration of nonweight-bearing following this procedure. According to the recent literature, given the costs and risks associated with revision surgery, it may be prudent to use locking plates in combination with lag screws and to maintain at least two weeks of nonweight-bearing. Emerging data on the use of locking plates appear promising, with current evidence suggesting lower complication rates. External fixation can offer excellent stability and may allow earlier weight-bearing; however, the management and potential complications of external devices remain concerns for both surgeons and patients. Innovative fixation methods, such as the use of arthroscopy for this procedure, are relatively recent developments and currently lack sufficient published evidence to support their widespread adoption. Regardless of the fixation technique selected, careful joint preparation and adequate compression are essential to minimizing the risk of failure in first TMTJ fusion.

## Figures and Tables

**Table 1:** Indications and contraindications for the Lapidus procedure.

**Indications**	
Frequent	Moderate to severe HV *(IMA > 15)* Hypermobility of the first ray *(in relation to the lesser metatarsal and/or plantar gapping of I TMTJ on lateral X-rays)* HV deformity with Pes planovalgus Generalized hyperlaxity *(Beighton score > 5)*
Rare	Recurrent HV or salvage procedure for failed bunion surgery *(satisfaction rate 80%)* Juvenile HV *(only when growth plate is closed)* I TMTJ osteoarthritis (*degenerative or posttraumatic)* Comminuted fractures of the I TMT
**Contraindications**	
Absolute	Acute or chronic infection with or without osteomyelitis. Juvenile HV (*open epiphysis of the first metatarsal base)* Degenerative arthritis of the I TMTJ Charcot arthropathy of the midfoot (*with or without poorly controlled diabetes)*
Relative	Smoking Osteoarthritis of the adjacent joints *(naviculocuneiform)* Preexisting short first metatarsal (*consider bone block interposition)* (Elite) athletes (*consider first joint-preserving procedures)*

## Data Availability

The contributions presented in this study are included in the article and in the cited ones. Further inquiries can be directed to the corresponding author.

## Abbreviations

The following abbreviations are used in this manuscript:

HV	Hallux valgus
TMTJ	Tarsometatarsal joint
HVA	Hallux valgus angle
IMA	Intermetatarsal angle
PROMs	Patient-reported outcome measures
CORA	Center of rotation of angulation
MTPJ	Metatarsophalangeal joint
DMMA	Distal metatarsal articular angle
IPA	Interphalangeal angle
TA	Tibialis anterior
EHL	Extensor hallucis longus
